# A Genomic Approach to Resolving Relapse versus Reinfection among Four Cases of Buruli Ulcer

**DOI:** 10.1371/journal.pntd.0004158

**Published:** 2015-11-30

**Authors:** Miriam Eddyani, Koen Vandelannoote, Conor J. Meehan, Sabin Bhuju, Jessica L. Porter, Julia Aguiar, Torsten Seemann, Michael Jarek, Mahavir Singh, Françoise Portaels, Timothy P. Stinear, Bouke C. de Jong

**Affiliations:** 1 Department of Biomedical Sciences, Institute of Tropical Medicine, Antwerp, Belgium; 2 Helmholtz Centre for Infection Research, GMAK, Braunschweig, Germany; 3 Department of Microbiology and Immunology, Doherty Institute for Infection and Immunity, University of Melbourne, Melbourne, Victoria, Australia; 4 Centre de Dépistage et de Traitement de l’Ulcère de Buruli Gbemotin, Zagnanado, Benin; 5 Victorian Life Sciences Computation Initiative, University of Melbourne, Parkville, Victoria, Australia; 6 New York University, New York, New York, United States of America; Fondation Raoul Follereau, FRANCE

## Abstract

**Background:**

Increased availability of Next Generation Sequencing (NGS) techniques allows, for the first time, to distinguish relapses from reinfections in patients with multiple Buruli ulcer (BU) episodes.

**Methodology:**

We compared the number and location of single nucleotide polymorphisms (SNPs) identified by genomic screening between four pairs of *Mycobacterium ulcerans* isolates collected at the time of first diagnosis and at recurrence, derived from a collection of almost 5000 well characterized clinical samples from one BU treatment center in Benin.

**Principal Findings:**

The findings suggest that after surgical treatment—without antibiotics—the second episodes were due to relapse rather than reinfection. Since specific antibiotics were introduced for the treatment of BU, the one patient with a culture available from both disease episodes had *M*. *ulcerans* isolates with a genomic distance of 20 SNPs, suggesting the patient was most likely reinfected rather than having a relapse.

**Conclusions:**

To our knowledge, this study is the first to study recurrences in *M*. *ulcerans* using NGS, and to identify exogenous reinfection as causing a recurrence of BU. The occurrence of reinfection highlights the contribution of ongoing exposure to *M*. *ulcerans* to disease recurrence, and has implications for vaccine development.

## Author Summary

We compared the whole genomes of four pairs of *Mycobacterium ulcerans* isolates collected at the time of first diagnosis and at recurrence, derived from a collection of almost 5000 well characterized clinical samples from one BU treatment center in Benin. Our findings suggest that after surgical treatment—without antibiotics—the second episodes were due to relapse rather than reinfection. Since specific antibiotics were introduced for the treatment of BU, the one patient with a culture available from both disease episodes had *M*. *ulcerans* isolates with a larger genomic distance, suggesting that the patient was most likely reinfected rather than having a relapse. To our knowledge, this study is the first to assess recurrences in *M*. *ulcerans* using whole genomes, and to identify exogenous reinfection as causing a recurrence of BU. The occurrence of reinfection highlights the contribution of ongoing exposure to *M*. *ulcerans* to disease recurrence, and has implications for vaccine development.

## Introduction

Buruli ulcer (BU) is a neglected necrotizing skin and bone disease caused by the enigmatic pathogen *Mycobacterium ulcerans* that occurs in riverine regions of West and Central Africa.

The clonal nature of *M*. *ulcerans* has complicated molecular analyses of the epidemiology of the pathogen, as genotyping methods with sufficient resolution have been lacking [1]. Using insertion sequence element single nucleotide polymorphism (ISE-SNP) typing, a technique in which two relatively small regions (1,431 and 1,871 bp) of the *M*. *ulcerans* genome are screened for polymorphisms, strains could be distinguished to the level of the river basin in West Africa [2]. However, such molecular genotyping techniques lack sufficient resolution to distinguish relapse from reinfection with an unrelated exogenous strain of *M*. *ulcerans* among BU recurrences. The World Health Organization (WHO) defines a relapse as a recurrence of BU within one year after termination of antibiotic treatment [3]. A recurrence that appears after that period is consequently considered a reinfection. The contribution of relapses versus reinfections to BU recurrences and their biological basis are to date unknown.

Until the routine use of antibiotics (rifampicin and streptomycin) was advocated by the WHO in 2004, surgery was the mainstay of BU therapy. After surgical treatment only, a recurrence rate of 6% was reported in Benin [4,5]. Higher recurrence rates were reported in Ghana (16%-35%) [6,7], Ivory Coast (17%) [8], Uganda (20%) [9], and Australia (32%) [10]. When specific antibiotics are used very few, if any, recurrences are observed [11–13].

The introduction of Next Generation Sequencing (NGS) now allows for the first time to distinguish relapses from reinfections in patients with multiple BU episodes. Similar studies have been conducted for *Mycobacterium tuberculosis* [14,15] and other monomorphic bacterial infections, such as *Clostridium difficile* [16]. In the present study we compared the number and location of SNPs identified by genomic screening between four pairs of *M*. *ulcerans* isolates collected at the time of first diagnosis and at recurrence, derived from a collection of almost 5000 well characterized clinical samples from one BU treatment center in Zagnanado, Benin, between 1989 and 2010.

## Methods

### Study design and participants

We defined an episode as a clinical suspicion of BU and a recurrence as the presence of two episodes separated by at least six months. Four such patients were identified for this study.

**Fig 1:**
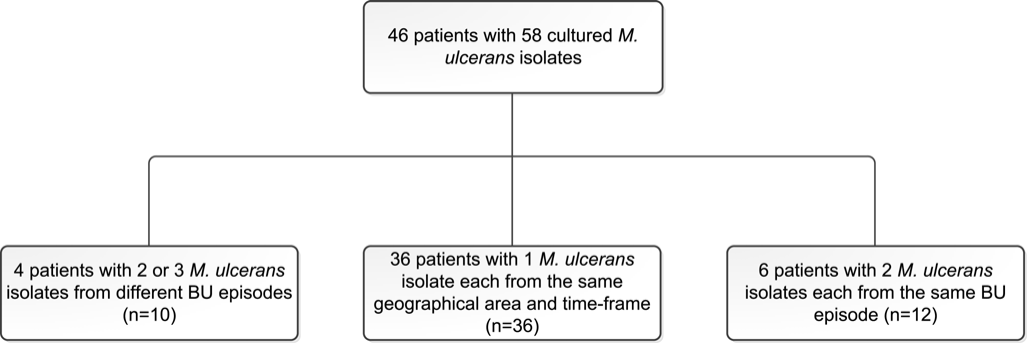
Flow chart outlining the *M*. *ulcerans* isolates included in this analysis.

We compared the number of SNP differences separating the paired isolates and a random selection of 36 isolates from 36 patients living in the same geographical area and diagnosed with BU in the same time frame (1998–2008) as the patients with multiple episodes. We also compared genomic relatedness between six patients with two *M*. *ulcerans* cultures isolated from the same disease episode. This genetic background helped to avoid misclassifying any second episodes with similar *M*. *ulcerans* strains prevalent in the patient’s environment as relapses rather than reinfections. As such a total of 58 *M*. *ulcerans* isolates obtained from 46 patients was included in this study (Fig 1).

### Genome sequencing and analysis

DNA was obtained by harvesting the growth of three Löwenstein-Jensen slants followed by heat inactivation, mechanical disruption, enzymatic digestion and DNA purification on a Maxwell 16 automated platform, a technique modified from Käser *et al*. [17].

Whole genome sequencing of the isolates was performed using an Illumina HiSeq 2000 DNA sequencer and an Illumina MiSeq DNA sequencer with Nextera XT or TruSeq (Illumina Inc., San Diego, CA, USA) library preparation and 2x36bp 2x100bp 2x150bp 2x250bp sequencing paired-end chemistry. Sequencing statistics are provided in S1 Table.

The sequencing data analysis was done using the Nesoni software [18]. Firstly, reads were filtered to remove those containing ambiguous base calls, any reads <50 nucleotides in length, and reads containing only homopolymers. All reads were furthermore trimmed removing residual ligated Nextera of TruSeq adaptors and low quality bases (<Q10) at the 3' end. Average read lengths after clipping are summarized for all isolates in S1 Table. Bowtie2 v2.1.0 [19] was used with default parameters to map clipped sequence reads sets to the Ghanaian *M*. *ulcerans* Agy99 reference genome (Genbank accession number: CP000325). Due to the unreliability of read mapping in repetitive regions, all ISE elements (IS*2404* and IS*2606*) were hard masked in this reference genome. Average read depths after mapping to *M*. *ulcerans* Agy99 are summarized for all isolates in S1 Table.

We compared the number and location of SNPs between isolates collected at baseline and at recurrence. At each of the loci called as a variant in any read set, Nesoni was used to generate a multi-way summary of consensus allele calls at the corresponding locus in all other read sets of the investigated panel. By concatenating all these loci a multiple SNP sequence alignment was generated containing all 282 variant loci across the Agy99 reference chromosome sequence. A maximum likelihood (ML) phylogenetic tree was constructed from this alignment using RAxML v8.0.19 [20] under a GTR model of evolution (no rate heterogeneity) and with an ascertainment bias likelihood correction for SNP data. The resulting tree was visualized in Figtree v1.4.0 [21] with nodes of interest highlighted. A haplotype network was derived using the median joining algorithm [22] as implemented within SplitsTree v.4.13.1 [23] with default settings. This network was subsequently visualized with Hapstar v.0.7 [24].

**Fig 2:**
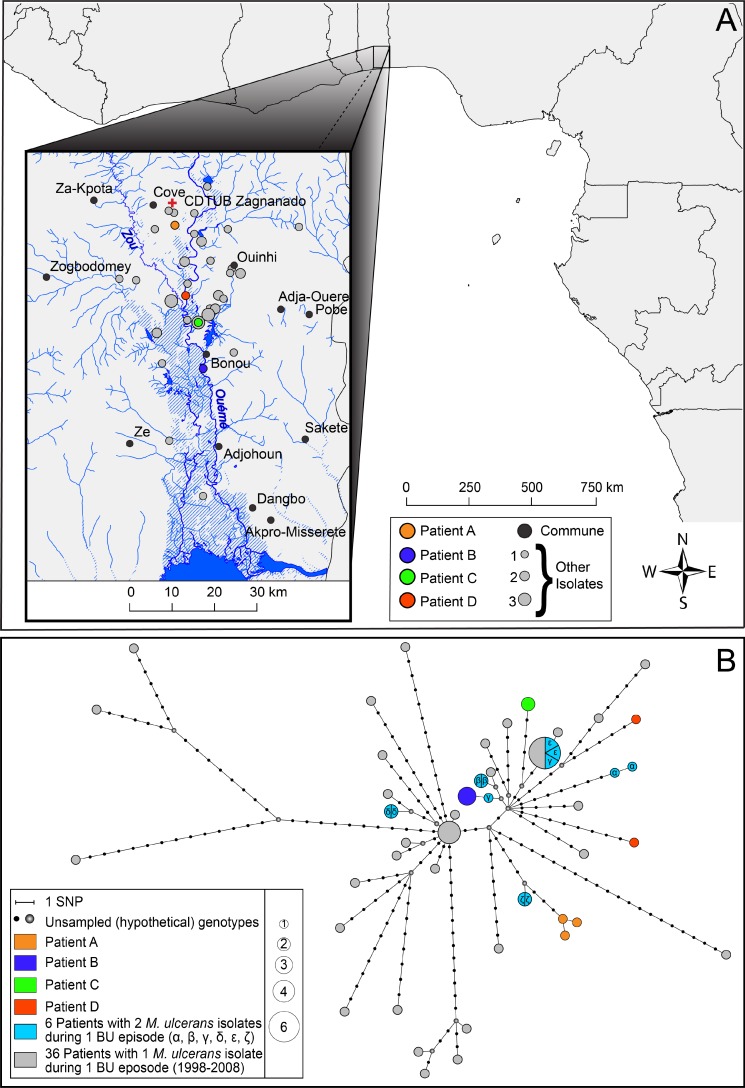
A: Geographical origin of the 58 isolates included in the study. Patients of special interest are colored accordingly. The size of the dots is proportional to the number of patients originating from a specific village varying from 1 to 3. The Ouémé river and its tributaries, and the BU treatment center of Zagnanado are shown as well. B: Median-Joining network showing patterns of descent among the 58 studied *M*. *ulcerans* isolates. Each circle represents a unique genotype, and the size of the circle is proportional to the number of individuals sharing that type. Every edge represents a single mutational step (or SNP) separating the sampled and hypothetical genotypes. Genotypes obtained from patients of special interest are colored accordingly.

The open source geographic information system Quantum GIS (QGIS v1.8.0) [25] was used to generate the illustration of the geographical distribution of all included *M*. *ulcerans* genomes in Fig 2. The geographical locations of the residences of BU patients at the time of their first consultation are shown. The river layer (Ouémé river and its tributaries) was digitized from declassified Soviet military topographic maps b31-03, b31-09, and b31-15 (scale 1:200k). The administrative borders of African countries were rendered from the Global Administrative Unit Layers data set of FAO [26].

### Ethics

This retrospective study on stored isolates was approved by the Institutional Review Board of the Institute of Tropical Medicine.

### Accession numbers

Read data for the study isolates have been deposited in the NCBI Sequence Read Archive (SRA) under accession n° PRJNA296792.

## Results

**Fig 3:**
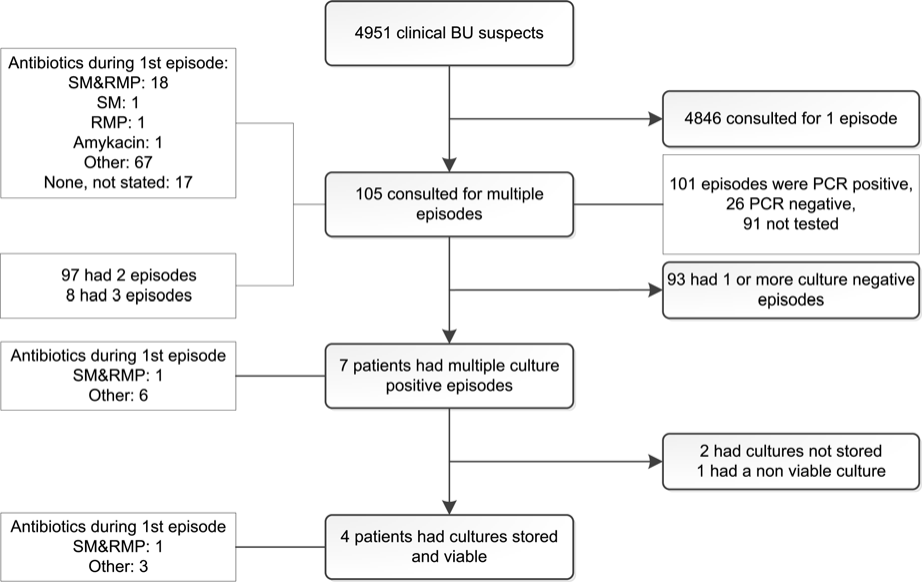
Flow chart outlining the patients contributing isolates to this analysis. SM: streptomycin; RMP: rifampicin.

Among the 4951 clinically BU suspected patients who consulted the BU treatment centre of Gbemotin in Zagnanado in southern Benin between 1989 and 2010, we identified 100 who presented with multiple BU episodes (Fig 3). A majority of 93 patients had two disease episodes while 7 had three episodes. Twenty recurrence patients received streptomycin and/or rifampicin during their first BU episode. The distribution of patients that had received (partially) effective antibiotics is shown in the S1 Fig. Only for seven of the 100 recurrence patients were we able to successfully culture isolates from each of two or three disease episodes, owing to the limited sensitivity of culture for isolation of *M*. *ulcerans* from skin biopsies [27]. These mycobacterial isolates were stored at ≤70°C in Dubos broth enriched with growth supplement and glycerol. However, paired cultures were found to be viable for only four of these seven patients. The first two patients of these four patients each had three paired isolates while the other two patients each had two (Table 1).

**Table 1:** Details on the four patients with recurrent BU.

ID		Patient A	Patient B	Patient C	Patient D
**Gender**		M	F	M	M
**Age at first consultation**		12y	9y	18y	10y
**Time between two episodes**		9.5 months	7 months	22,5 months	9 months
**Episode 1**	**location**	right lower limb	left lower limb	left upper limb	left back/flank
	**date of presentation**	7-Apr-1999	10-Jul-2000	27-May-2002	1-Mar-2004
	**treatment**	PCN GM	CXA GM	CXA GM	CXA SM RMP
		surgery	surgery	surgery	surgery
	**cultures stored**	1	1	1	1
		ITM992421	ITM001249	ITM021902	ITM041698
**Episode 2**	**location**	right lower limb	left lower limb	left lower limb	right buttock
	**cultures stored**	2	2	1	1
		ITM000562	ITM010548	ITM041716	ITM051516
		ITM000563	ITM010623		
**SNP difference**		1	0	0	20

PCN: penicillin; GM: gentamycin; CXA: cloxacillin, SM: streptomycin, RMP: rifampicin.

We assessed the paired *M*. *ulcerans* isolates of four patients (letter-coded from A to D) with two BU episodes each (Table 1). Each of these 4 patients presented for the first time at the BU treatment centre of Gbemotin between 1999 and 2004. Patients A & B had lesions at the same location during both episodes, while patients C & D had lesions at another body site. The time between the diagnoses of both episodes ranged from seven months to almost two years. All patients underwent surgery, while patient D also received specific antibiotics, which were introduced for the routine management of BU in 2004 [3]. Comparative genomic analysis identified two patients (B & C) with no detectable genetic differences (0 SNPs) between isolates originating from two disease episodes. Patient A had one SNP difference between his first episode isolate and both second episode isolates although at different positions, suggesting that micro-evolution took place. For patient D however the isolates of both disease episodes were differentiated from each other by no less than 20 SNPs, which were found distributed throughout the genome. This is a genetic distance similar to that observed between different isolates from the same geographic region as illustrated in the haplotype network (Fig 2B) and the phylogenetic tree (red nodes, S2 Fig). The difference in SNPs between control patients varied from 0 to 53 SNPs. To exclude cross-contamination of the *M*. *ulcerans* culture obtained from the second episode of patient D, the genome obtained from a strongly positive biopsy that was treated on the same day as the biopsy of patient D was sequenced as well and found to differ by 21 SNPs.

There were 2 clusters of 5 and 4 patients having identical *M*. *ulcerans* genomes, who lived in an area of respectively 70 km² (4.7–27 km between villages) and 14 km² (3–18 km between villages). Four patients with multiple *M*. *ulcerans* isolates from the same BU episode had identical paired genomes while one such patient differed in 1 SNP and another one in 6 SNPs between the paired genomes with respectively 8 and 7 days between the times of sampling of the biopsies from which the *M*. *ulcerans* cultures were isolated (Fig 2B).

Among the total of 24 substitutions that were identified in patient A and patient D, four occurred in intergenic regions, while 20 were found in coding sequences resulting in 3 synonymous changes (i.e. ‘silent’ changes) and 17 non-synonymous changes (i.e. resulting in a change in amino acid), in genes encoding proteins with various functions (S2 Table).

## Discussion

This study is the first to study recurrences in *M*. *ulcerans* using NGS, and to identify exogenous reinfection as causing a recurrence of BU. In relapses, paired isolates are genetically more related to each other than to isolates from other patients living in the same region and infected during the same time-period. In reinfections on the other hand, paired isolates are potentially not more related to each other than to isolates from other patients living in the same region and infected during the same time-period.

Our results suggest that the second BU episode of patients A, B and C was most likely due to relapses. The second BU episode of the 4th patient was probably due to a reinfection. This patient is also the only one who had received specific antibiotics during his first BU episode. We can however not be entirely certain that patients A, B and C were not infected a second time by an identical *M*. *ulcerans* strain, as we identified other genetically identical clusters among patients living in the same area and time period. As the mode of transmission of *M*. *ulcerans* remains enigmatic, detailed investigation of these genetic clusters may provide leads to a common point source of exposure.

To our knowledge, this study is the first using NGS to assess recurrences in *M*. *ulcerans*, which is important to understand BU epidemiology. In BU, when disease re-occurs within one year after the end of treatment it is assumed to be a relapse which is considered the primary endpoint in several studies [11–13] and in patient management. This study for the first time indicates that exogenous reinfection plays a role in recurrence of BU. However, the restricted phylogeny presented here based on four patients should be interpreted with some circumspection because of the limited sample size, which is due to the overall low recurrence rate at this treatment center, combined with the low sensitivity of *in vitro* culture of *M*. *ulcerans*.

In other mycobacterial infections, most notably infection with *M*. *tuberculosis*, the number of different SNPs between relapse and reinfection pairs can be large, with a clear distinction between pairs with a small difference (≤6 SNPs), classified as probably relapses, and those with a large difference (≥1306 SNPs), deemed to be reinfections [15]. Epidemiologically linked *M*. *tuberculosis* populations have been reported with a mean SNP difference of 3.4 demonstrating high genomic stability [28]. Walker and colleagues [29] reported that in patients with an epidemiological link the divergence between their *M*. *tuberculosis* genomes generally does not exceed 14 SNPs, with most patients having fewer than 5 SNP differences during one disease episode. During recurrences of *Clostridium difficile* infection, paired isolates ≤2 SNPs apart were considered relapses while paired isolates >10 SNPs apart were considered reinfections [16]. In the present study we detected only one genomic difference between the relapse pairs, reaffirming the high genomic stability previously reported for *M*. *ulcerans* [1].

Apparent reinfections could theoretically result from differential sampling of an initially mixed infection. Patient D could possibly have had a simultaneous infection by two *M*. *ulcerans* strains although the results from the 6 patients with multiple isolates from one BU episode suggest that different *M*. *ulcerans* strains causing a single disease episode is, at best, an infrequent occurrence. The same was observed among four Cameroonian BU patients with multiple isolates from one BU episode [30]. This suggests mixed infection would have been an unlikely explanation for the genetic differences between the paired isolates of patient D.

The occurrence of reinfection highlights the contribution of ongoing exposure to *M*. *ulcerans* to disease recurrence. Since the delay between relapse (7, 9.5, and 22.5 months) and reinfection (9 months) episodes overlap, the use of NGS on cultured isolates is required to distinguish these two scenarios. BU recurrences within a period of one year after antibiotic treatment that are considered relapses by WHO [3] may therefore also be reinfections.

Since BU transmission is probably not human-to-human and the mode of transmission from the environment is not yet clarified, epidemiological links in support of transmission routes are speculative at best. However, we expect to be able to determine a SNP-threshold which can be interpreted as an epidemiological link consistent with a common source of infection in an ongoing study with a greater number of *M*. *ulcerans* genomes from the Ouémé river valley. The definition of transmission clusters could help to unravel the enigmatic transmission routes of *M*. *ulcerans*.

NGS of paired *M*. *ulcerans* strains collected from patients with multiple episodes of BU has sufficient resolution to distinguish relapse from reinfection. Our results on a small number of patients suggest that after surgical treatment without antibiotics the second episodes were due to relapse rather than reinfection. Since specific antibiotics were introduced for the treatment of BU, the only patient with a culture available from both disease episodes had *M*. *ulcerans* isolates with a greater genetic distance, suggesting this patient was most likely reinfected.

## Supporting Information

S1 Fig Proportion of clinical suspects that had either not received any specific antibiotics or (partially) effective antibiotics (rifampicin, streptomycin, amikacin or clarithromycin) during their first disease episode stratified by delay between episodes (6 months: cutoff in this study; 12 months: WHO cutoff). (TIF)

S2 Fig Unrooted phylogenetic SNP tree: RAxML was used to build a maximum likelihood tree from the genomic SNP data with 282 variable positions among 58 genomes. Nodes of interest are colored according to subject. The scale indicates expected number of substitutions per site.
(TIF)

S1 Table Sequencing statistics of the study isolates. (XLSX)

S2 Table Description of substitutions in patients A and D. (XLSX)
