# Supplementary figures and images for: A Genomic Approach to Resolving Relapse versus Reinfection among Four Cases of Buruli Ulcer
Source: PLoS Negl Trop Dis. 2015 Nov 30;9(11):e0004158. doi: 10.1371/journal.pntd.0004158 (PMC4664471; doi:10.1371/journal.pntd.0004158)

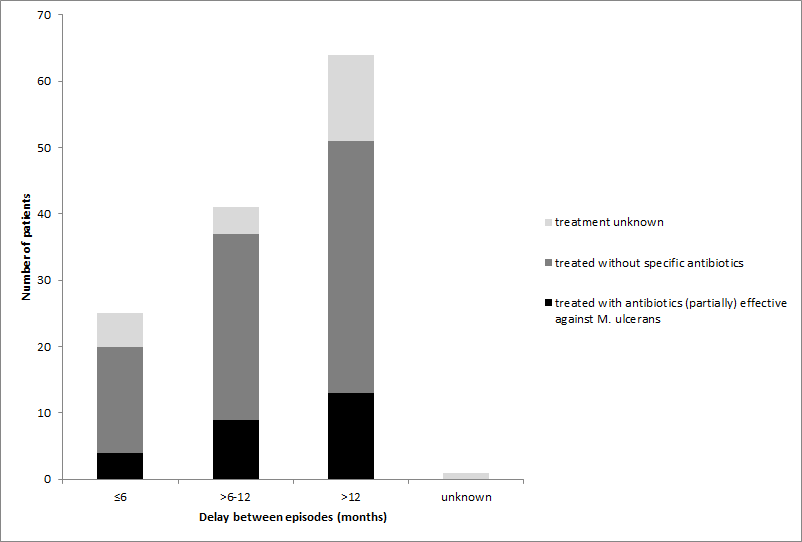

Supplement: S1 Fig — (TIF) [file pntd.0004158.s001.tif]

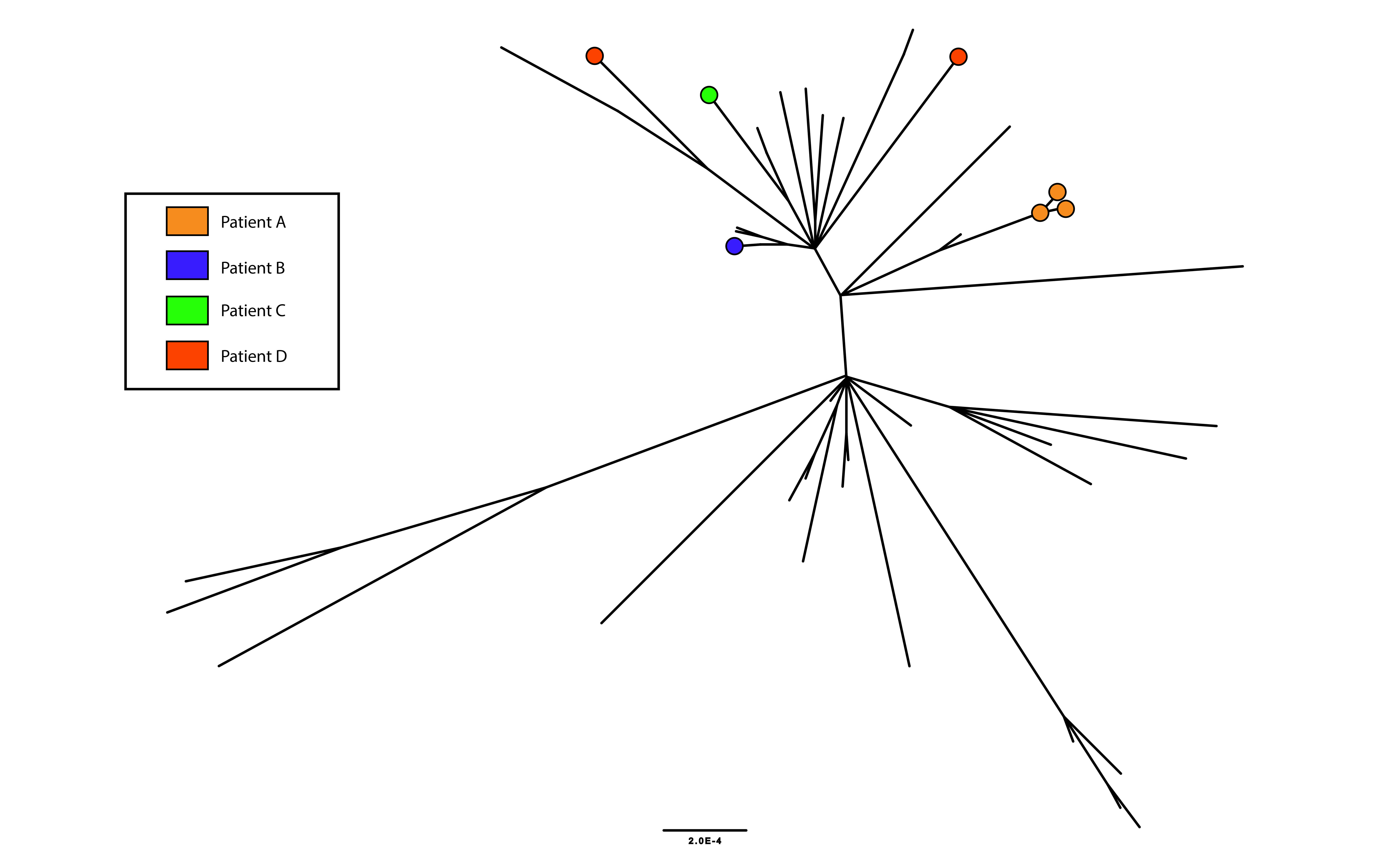

Supplement: S2 Fig — Nodes of interest are colored according to subject. The scale indicates expected number of substitutions per site. (TIF) [file pntd.0004158.s002.tif]
